# Nitric oxide promotes adventitious root formation in cucumber under cadmium stress through improving antioxidant system, regulating glycolysis pathway and polyamine homeostasis

**DOI:** 10.3389/fpls.2023.1126606

**Published:** 2023-03-09

**Authors:** Lijuan Niu, Yunlai Tang, Bo Zhu, Zhenfu Huang, Dan Wang, Qiyang Chen, Jian Yu

**Affiliations:** School of Life Science and Engineering, Southwest University of Science and Technology, Mianyang, Sichuan, China

**Keywords:** nitric oxide, cadmium, rooting response, antioxidants, glycolysis, polyamine pathway

## Abstract

Cadmium (Cd) as a potentially toxic heavy metal that not only pollutes the environment but also interferes with plant growth. Nitric oxide (NO) regulates plant growth and development as well as abiotic stress response. However, the mechanism underpinning NO-induced adventitious root development under Cd stress remains unclear. In this study, cucumber (*Cucumis sativus* ‘Xinchun No. 4’) was used as the experimental material to investigate the effect of NO on the development of adventitious roots in cucumber under Cd stress. Our results revealed that, as compared to Cd stress, 10 μM SNP (a NO donor) could considerably increase the number and length of adventitious roots by 127.9% and 289.3%, respectively. Simultaneously, exogenous SNP significantly increased the level of endogenous NO in cucumber explants under Cd stress. Our results revealed that supplementation of Cd with SNP significantly increased endogenous NO content by 65.6% compared with Cd treatment at 48 h. Furthermore, our study indicated that SNP treatment could improve the antioxidant capacity of cucumber explants under Cd stress by up-regulating the gene expression level of antioxidant enzymes, as well as reducing the levels of malondialdehyde (MDA), hydrogen peroxide (H_2_O_2_) and superoxide anion ( ${\text{ O}}_{2}^{\;\cdot -}$ ) to alleviate oxidative damage and membrane lipid peroxidation. Application of NO resulted in a decrease of the ${\text{ O}}_{2}^{\;\cdot -}$ , MDA, and H_2_O_2_ level by 39.6%, 31.4% and 60.8% as compared to Cd-alone treatment, respectively. Besides that, SNP treatment significantly increased the expression level of related genes involved in glycolysis processes and polyamine homeostasis. However, application of NO scavenger 2-(4-carboxy -2-phenyl)-4, 4, 5, 5-tetramethy limidazoline -1-oxyl -3-oxide (cPTIO) and the inhibitor tungstate significantly reversed the positive role of NO in promoting the adventitious root formation under Cd stress. These results suggest that exogenous NO can increase the level of endogenous NO, improve antioxidation ability, promote glycolysis pathway and polyamine homeostasis to enhance the occurrence of adventitious roots in cucumber under Cd stress. In summary, NO can effectively alleviate the damage of Cd stress and significantly promote the development of adventitious root of cucumber under Cd stress.

## 1 Introduction

Cadmium (Cd), a widely spread heavy metal, is easily absorbed by plant roots, thus enters the food chain, and eventually poses a substantial threat to human health (Falco et al., 2005; Ahmad et al., 2016a; Ahmad et al., 2016b). It has been discovered that Cd, as a non-essential element for plant growth and development, disturbs nutrient and water uptake/transport (Rivelli et al., 2014; Hafsi et al., 2022). Moreover, Cd induces a number of stress responses in plants including ion balance changes (Kucerova et al., 2020; Hafsi et al., 2022), change in antioxidant enzymes activities (Guo et al., 2019), photosynthesis inhibition (Rizwan et al., 2018), and changes in the expression of related genes and proteins (Muhammad et al., 2019; Manara et al., 2020). Plant response to abiotic stress is usually accompanied by an increase in the level of reactive oxygen species (ROS) (Polle and Schützendübel, 2003; Kohli et al., 2019; Mansoor et al., 2022), and the increase in ROS content caused the destruction of cell structure and function (Panyuta et al., 2016; Kohli et al., 2019; Mansoor et al., 2022). Previous research have demonstrated that Cd stress could trigger the ROS generation, such as hydrogen peroxide (H_2_O_2_) and superoxide radical ( ${\text{ O}}_{2}^{\;\cdot -}$ ) accumulation in plants (Qi et al., 2021; Li et al., 2022). Some studies have also shown that excessive accumulation of ROS under Cd stress can trigger protein post-translational modification (Gzyl et al., 2015), enzyme inactivation and denaturation, DNA and RNA damage, resulting in cell damage and cell death (Singh et al., 2016). These series of reactions may aggravate the degree of lipid peroxidation (Heyno et al., 2008), disrupts metabolic activities and eventually affect plant growth and development (Yu et al., 2015; Anwar et al., 2021; El Rasafi et al., 2022). It has been demonstrated that plants have a series of antioxidant defense system to mitigate the oxidative damage caused by ROS (Gill and Tuteja, 2010). Antioxidant enzymes such as ascorbate peroxidase (APX), superoxide dismutase (SOD), catalase (CAT) or glutathione reductase (GR) have been demonstrated to regulate accumulation of ROS and protect plants from oxidative damage under Cd stress (Irfan et al., 2014; Guo et al., 2019). Therefore, the possibility of oxidative signal or oxidative damage depends on the balance between antioxidant enzyme activity and ROS level (Møller et al., 2007).

Nitric oxide (NO) has been implicated as an essential signaling molecule in plants. Numerous studies have discovered that NO plays an essential role in the regulation of plant growth and development including seed germination (Ren et al., 2020), root growth and development (Pagnussat et al., 2002; Sun et al., 2019; Liu et al., 2022a), pollen tube germination (Prado et al., 2004) and fruit senescence (Zuccarelli et al., 2021). The increasing evidence indicates that, NO function in plant stress response. As a multi-functional regulator, NO signaling is involved in a range of abiotic stress responses to mitigate oxidative damage caused by abiotic stress (Parankusam et al., 2017; Gao et al., 2022; Xia et al., 2022). For example, exogenous NO could stabilize the cell membranes in hulless barley under drought stress (Gan et al., 2015). Moreover, application of NO could upregulate the gene expression of antioxidative enzymes to enhance the antioxidant capacity under Cd stress (Chen et al., 2010).Thus, the protective roles of NO in alleviating oxidative injury have focused on regulating antioxidant systems, reducing the generation of ROS, mediating related gene expression, and maintaining protein stability, eventually enhancing plant stress tolerance (Terrón-Camero et al., 2019; Wei et al., 2020).

Cucumber is a member of the Cucurbitaceae family (Hashem et al., 2018). As one of the most popular vegetables, cucumber is shallow-rooted crop and is used to be an bioindicator species to assess toxicity of soils polluted by Cd (An et al., 2004). As mentioned above, NO plays an essential role in regulating plant growth and development. Moreover, it has been shown that NO is involved in the response to Cd stress. However, the mechanism underpinning NO-induced adventitious root development in cucumber under Cd stress remains unclear. The aim of this study was to investigate the role of NO in promoting the development of adventitious root in cucumber under Cd stress. Therefore, we conduct this experiment to test the effect of NO on root development, oxidative defence, glycolysis and polyamine metabolism in cucumber under Cd stress. The objective of this study was to provide evidence to elucidate the potential mechanism of NO signaling in responses to Cd stress in plants.

## 2 Materials and methods

### 2.1 Plant materials

*Cucumis sativus* L. (‘Xinchun No. 4’) was used in this experiment. The sterilized cucumber seeds were pre-soaked in distilled water for 5 hours. The seeds were germinated on filter paper in petri dishes and then incubated in a climate box at 25°C with a 14 h photoperiod (200 μmols^-1^m^-2^). The experiment was repeated three times, with 10 seedlings per replicate.

### 2.2 Explant treatments

**Experiment 1**: Sodium nitroprusside (SNP, purity≥98.5%, Solarbio, China) as a NO donor. Cucumber explants were placed in petri dishes containing distilled water or different concentrations of SNP (0, 1, 10, 100, 500 μM) under Cd stress for 5 days. The concentrations of NO was selected based on the results of our previous studies (Niu et al., 2017; Niu et al., 2019). These media were changed every day in order to keep the solution fresh.

**Experiment 2:** 200 μM 2-(4-carboxy-2-phenyl)-4, 4, 5, 5-tetramethylimidazoline -1-oxyl-3-oxide (c-PTIO, purity≥98%, Sigma, USA) as NO scavenger, 200 μM tungstate (Solarbio, China) as a NO inhibitor. The concentrations of CdCl_2_, NO scavenger or inhibitor were based on the results of a preliminary experiment.

### 2.3 Endogenous NO content

The NO content was determined using the Greiss reagent method with minor modifications (Xuan et al., 2012). Cucumber explants were ground and mixed with 4 mL of 50 mM ice cold acetic acid buffer (containing 4% zinc diacetate). The mixture was centrifuged at 10000 *g* for 15 min at 4°C, and the supernatant was collected. Then, 0.1 g of charcoal was added. After vortex and filtration, the filtrate was mixed with 1mL Greiss reagent at room temperature for 30 min. Finally, the absorbance was assayed at 540 nm.

### 2.4 Malondialdehyde, superoxide anion ( ${\text{ O}}_{2}^{\;\cdot -}$ ) and hydrogen peroxide (H_2_O_2_) content

For measuring MDA, 0.2 g of samples were ground in ice bath and extracted with 5 mL trichloroacetic acid (TCA). The homogenate was transferred to a centrifuge tube and centrifuged at 4°C at 12000 *g* for 15 min. The supernatant was added to 0.5% TBA solution. The mixture is heated in a boiling water bath for 30 min and then centrifuged for 10 min. The absorbance of the supernatant was measured at 450 nm, 532 nm and 600 nm (Liu et al., 2022b). For estimating ${\text{ O}}_{2}^{\;\cdot -}$ generation, the samples were homogenized with potassium phosphate buffer (pH 7.8) and centrifuged for 10 min. The supernatant was added to hydroxylamine hydrochloride and reacted at 25°C for 20 min. Finally, the absorbance was measured at 530 nm (Gong et al., 2014). Superoxide accumulation was also examined by nitroblue tetrazolium (NBT) staining, as described previously (Wang et al., 2019). H_2_O_2_ content in cucumber explants was determined as described by the method with minor modification (Liao et al., 2011). 0.5 g of cucumber explants were ground in liquid nitrogen and then homogenized in 3 mL ice-cold aceton. After centrifugation at 10000 *g* for 10 min at 4°C, the reaction mixture composed of 0.5 mL of the supernatant, 0.5 mL of trichloromethane (CHCl_3_), 1.5 mL carbon tetrachloride (CCl_4_) and 2.5 mL of distilled water. The mixture was then centrifuged at 1000 *g* for 1 min and the supernatant fractions were collected for H_2_O_2_ determination. In addition, H_2_O_2_ was detected with the DAB method with some modifications. Briefly, leaves were placed in the diaminobenzidine (DAB) staining solution. After then, the treated leaves were placed in 95% ethanol for 10 min. The reaction of DAB with H_2_O_2_ could produce the deep brown polymerization product (Yang et al., 2013).

### 2.5 Quantitive real-time PCR assays

In order to investigate the effect of NO on antioxidant system, glycolysis and polyamine pathway during adventitious rooting under Cd stress. The relative expression of genes encoding for antioxidant enzymes, glycolysis pathway and polyamine biosynthetic enzymes were measured. Cucumber explants were ground into powder with liquid nitrogen. Total RNA was extracted using the DP419 kit (TianGen, Beijing, China). Quantitative real-time PCR reactions were performed using SYBR Green SuperReal PreMix Plus kit (TianGen, Beijing, China) according to the cycling parameters: 95°C for 15 min; 95°C for 10 s and 60°C for 32 s, 40 cycles. qRT-PCR amplification primers are shown in **Table 1**. The relative expression of the gene was calculated by the 2 ^-ΔΔCT^ method.

**Table 1:** Sequences of primers used for this study.

Gene	Forward Primer	Reverse Primer
Actin	5’-TTGAATCCCAAGGCGAATAG-3’	5’-TGCGACCACTGGCATAAAG-3’
*CsPOD*	5’-TTGTGATGGGTCGGTGCTAC-3’	5’-TGTCCTGATGCCAAGGTGAC-3’
*CsCAT*	5’- CATGGACGGTTCAGGTGTCA-3’	5’- CCACTCAGGGTAGTTGCCAG-3’
*CsAPX*	5’-CTGCTACTGTTTTTGGAACCGCCG-3’	5’- GCGGAGGAGAGGAAACGAGTAGTT-3’
*CsSOD*	5’-CACCCAAGAAGGAGACGGTC-3’	5’- CAGCAGGGTTGAAATGTGGC-3’
*CsGR*	5’-GATATGAGAGCCGTGGTTGC-3’	5’-AGTCGCAAACAACACAGCAT-3’
*CsPFK*	5’-TTGGTTGATAATTGGCATAAG-3’	5’-GCATCCACTATCTTCTTCA-3’
*CsPK*	5’-TGCTGTCATCACCTATTG-3’	5’-ACAAGAGTCGGTTTACAC-3’
*CsFK*	5’-CCTGGATGAAGAATACTATGA-3’	5’-CGGCGTGTAATGATAATG-3’
*CsHK*	5’-TGTTGTGGTGAAGTTCTT-3’	5’-CCTCCATTTCCCTCTATTC-3’
*CsADC*	5’-GGATCCCAGATCCCTTCTAC-3’	5’-GTCAATACCCAGACCACCTC-3’
*CsODC*	5’-CGTCGTTGGCGTGTCATTT-3’	5’- CAAGTCGGACTGCCGTTTC-3’
*CsPAO*	5’-TCTCCTTCTCGTTCCTCCGT-3’	5’-CCACCGACTCCAACAATCCA-3’

### 2.6 Statistical analysis

Three independent replicates were set for each experiment. Means were separated by Duncan test at 0.05 probability level. Analysis of variance (ANOVA) was done. SPSS V. 13.0 was used for statistical analysis.

## 3 Results

### 3.1 Effect of exogenous NO on adventitious root formation under Cd stress

**Figure 1:**
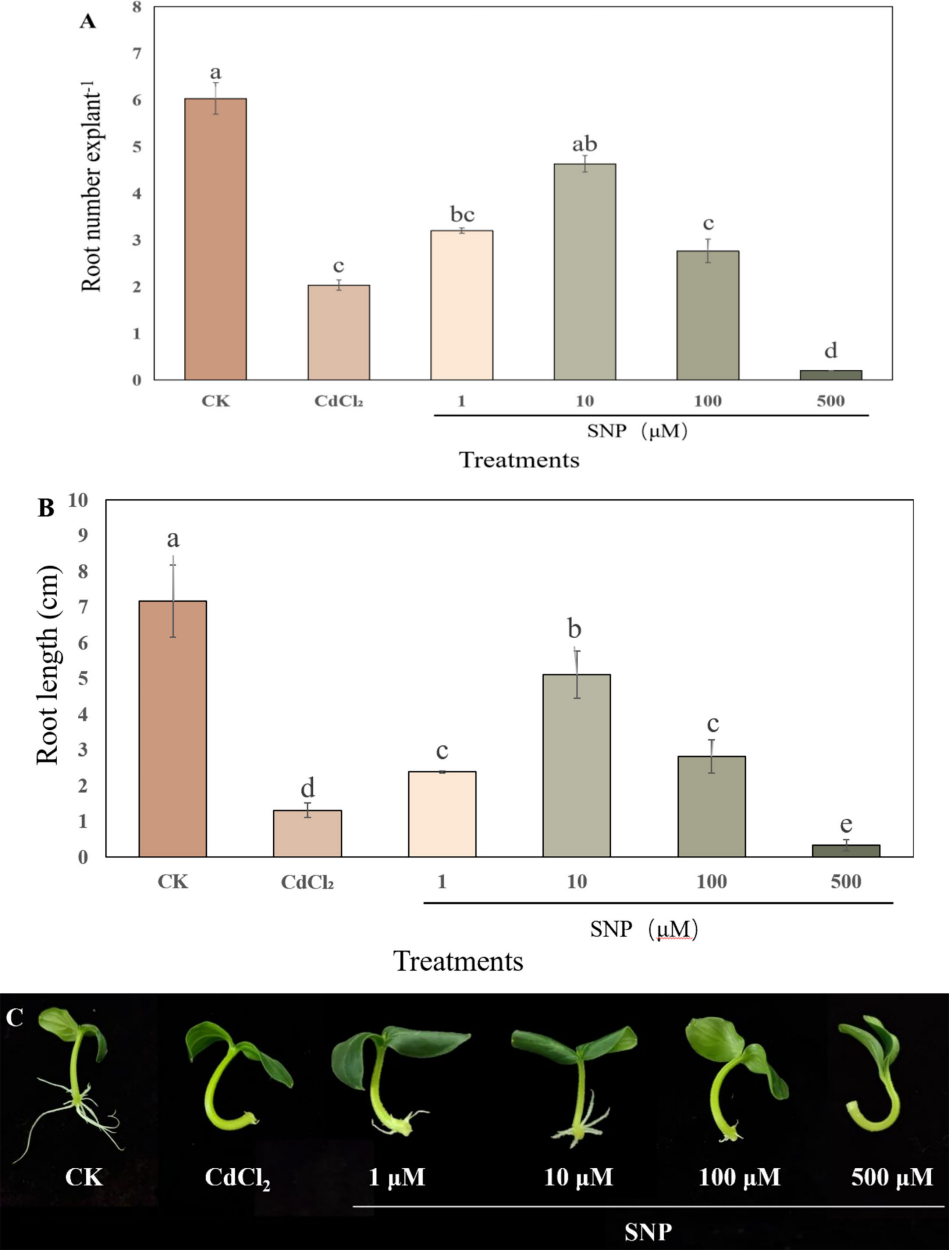
Effect of NO on adventitious root formation under Cd stress. The primary roots were removed from 5-day-old seedlings. Explants were then incubated for 5 days with distilled water (CK) or 1 μM CdCl_2_, 1 μM CdCl_2_ +1 μM SNP, 1 μM CdCl_2_ + 10 μM SNP, 1 μM CdCl_2_ + 100 μM SNP, 1 μM CdCl_2_ + 500 μM SNP. Ten explants were used per replicate. The numbers **(A)** and root length **(B)** of adventitious roots were expressed as mean ± SE (n = 3). Photographs **(C)** were taken after five days of the treatments indicated. Bars with different letters are significantly different at *P* < 0.05 according to Duncan’s multiple range test.

To understand the effect of exogenous NO on the development of adventitious root under Cd stress, we performed a dose-response experiment with NO. As shown in **Figure 1**, compared to CK treatment, CdCl_2_ treatment significantly reduced root number and root length by 66.3% and 81.7%, respectively. Moreover, the development of adventitious roots altered considerably with increasing concentrations of NO donor, SNP (1, 10, 100, 500 μM). As shown in **Figure 1**, the root number and root length of 10 μM NO treatment was significantly increased by 127.9% and 289.3%, respectively, as compared to CdCl_2_ treatments. However, a high concentration of NO (500 μM) obviously decreased the number and length of adventitious roots under Cd stress (**Figures 1A, B**). Therefore, exogenous NO displayed a concentration-dependent influence on adventitious rooting under Cd stress, and these results indicated that 10 μM NO significantly ameliorated the adverse effect of Cd stress on the development of adventitious roots.

### 3.2 Effect of cPTIO or tungstate on adventitious root formation under Cd stress

**Figure 2:**
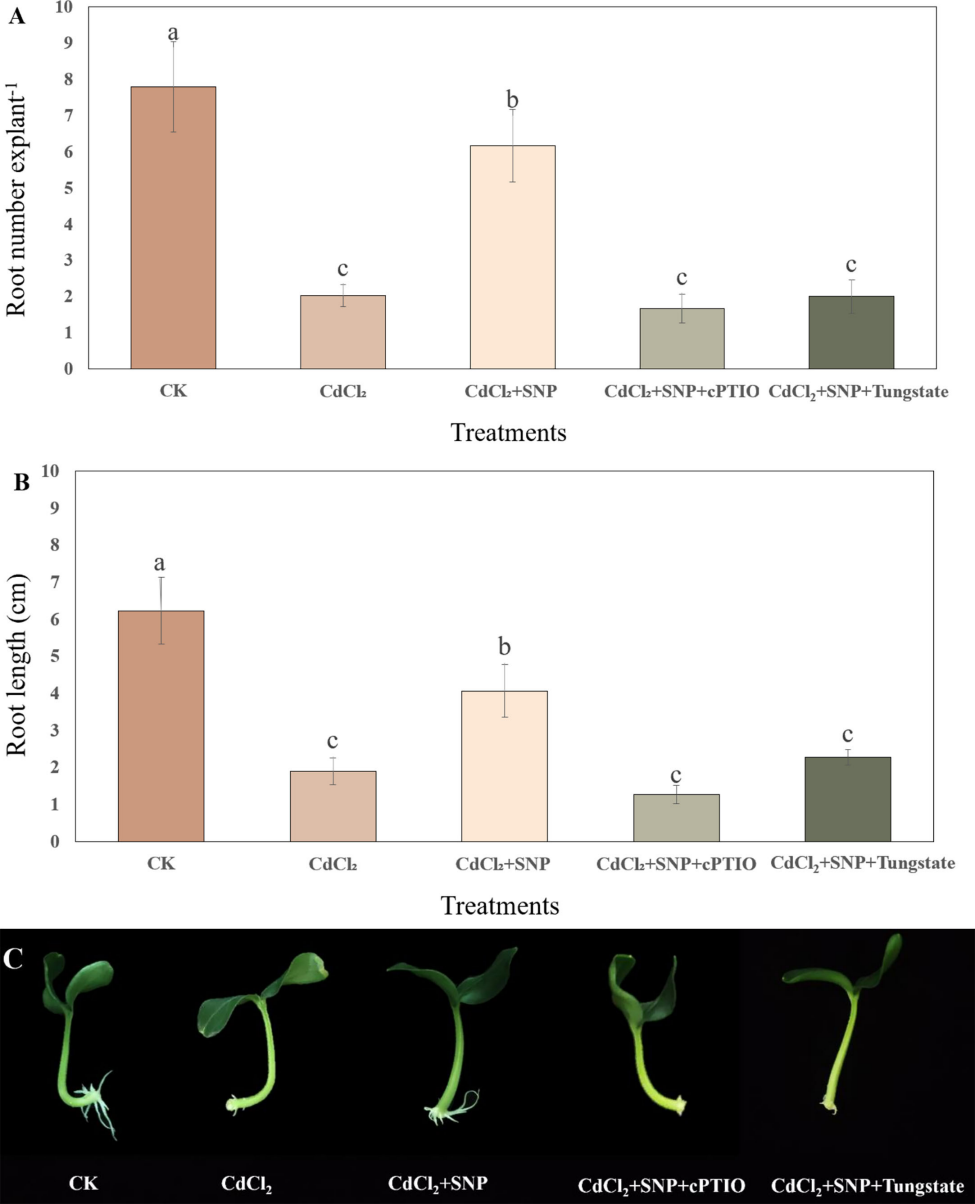
Effect of cPTIO or tungstate on adventitious root formation under Cd stress. The primary roots were removed from 5-day-old seedlings. Explants were then incubated for 5 days with distilled water (CK) or 1 μM CdCl_2_, 1 μM CdCl_2_ +10 μM SNP, 1 μM CdCl_2_ + 10 μM SNP + 200 μM cPTIO, 1 μM CdCl_2_ + 10 μM SNP + 200 μM tungstate. Ten explants were used per replicate. The numbers **(A)** and root length **(B)** of adventitious roots were expressed as mean ± SE (n = 3). Photographs **(C)** were taken after five days of the treatments indicated. Bars with different letters are significantly different at *P* < 0.05 according to Duncan’s multiple range test.

To further investigate the key role of NO in affecting adventitious root formation in cucumber under Cd stress, NO scavengers or inhibitor was utilized in this experiment. As shown in **Figure 2**, NO treatment obviously induced the adventitious rooting under Cd stress. However, application of cPTIO or tungstate significantly inhibited the NO-promoted adventitious rooting under Cd stress. The number of adventitious roots which treated with NO scavenger or inhibitor decreased by 73% and 67.6%, respectively, when compared to CdCl_2_ + NO treatment (**Figure 2A**). Meanwhile, the adventitious root length of explants treated with cPTIO or tungstate reduced by 68.9% and 44.3%, respectively, as compared to that of NO treatment (**Figure 2B**). These results implied that NO might be responsible for promoting the formation of adventitious root under Cd stress.

#### 3.3 Changes in the endogenous NO level during NO-induced adventitious root formation under Cd stress

**Figure 3:**
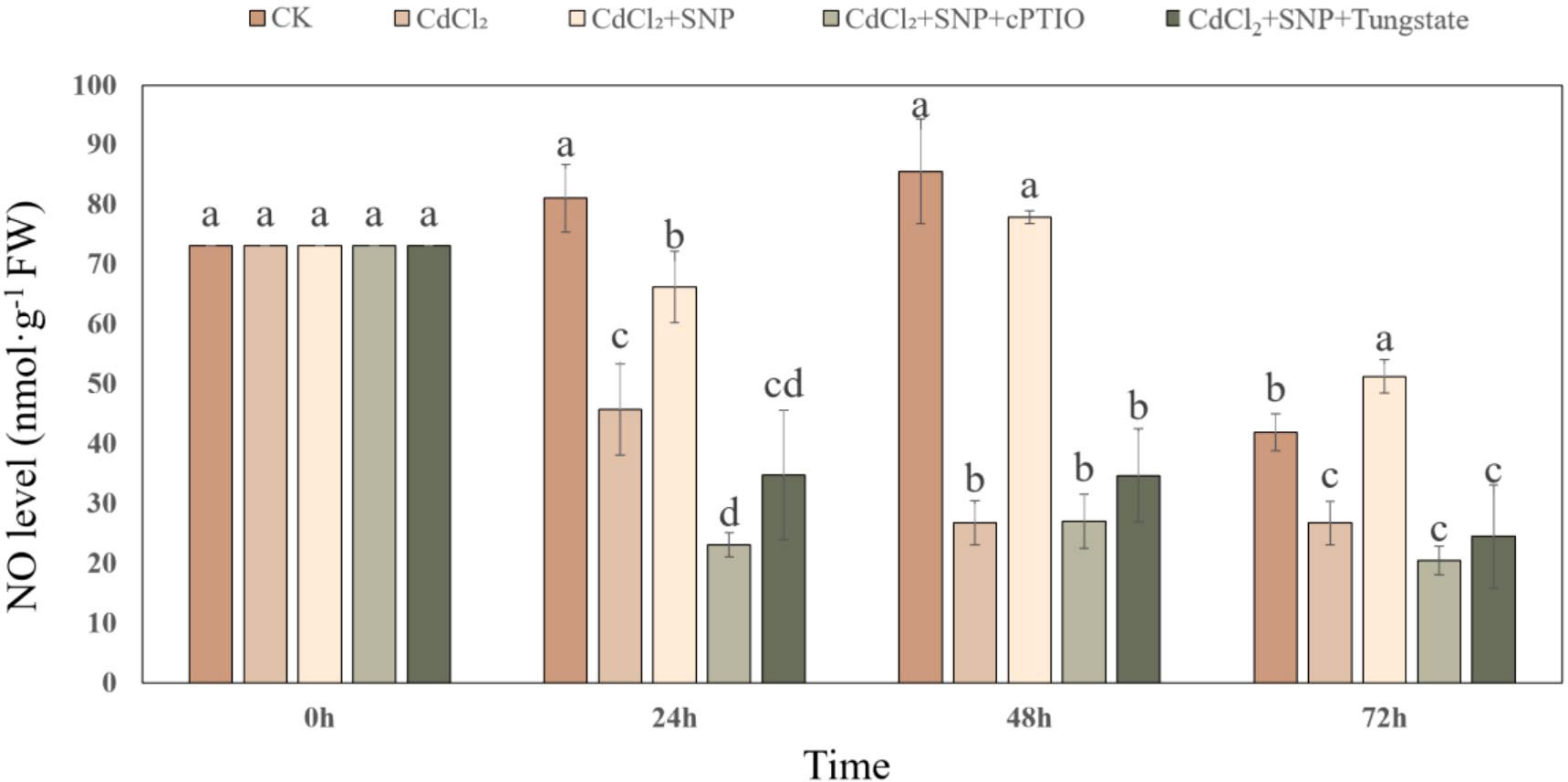
Changes in the endogenous NO level during NO-induced adventitious root formation under Cd stress. The primary roots were removed from 5-day-old seedlings. Explants were then incubated for 72 h with distilled water (CK) or 1 μM CdCl_2_, 1 μM CdCl_2_ +10 μM SNP, 1 μM CdCl_2_ + 10 μM SNP + 200 μM cPTIO, 1 μM CdCl_2_ + 10 μM SNP + 200 μM tungstate. Bars with different letters are significantly different at *P* < 0.05 according to Duncan’s multiple range test.

In order to further validate the influence of NO on adventitious root production under Cd stress, endogenous NO level was detected during NO-induced adventitious rooting under Cd stress condition (**Figure 3**). The concentration of endogenous NO in CdCl_2_ treatment gradually decreased during the process of adventitious root development (**Figure 3**). However, the level of endogenous NO which treated with CdCl_2_+NO was considerably higher than those in Cd group, reaching a maximum at 48 h. As shown in **Figure 3**, at 48 h, exogenous NO treatment significantly enhanced endogenous NO level by 65.6% as compared to CdCl_2_. However, cPTIO and tungstate treatments obviously decreased endogenous NO level during adventitious root development, which was 65.3% and 55.5% lower than that of NO treatment, respectively (**Figure 3**).

### 3.4 Effect of NO on reactive oxygen species during adventitious root development in cucumber under Cd stress

**Figure 4:**
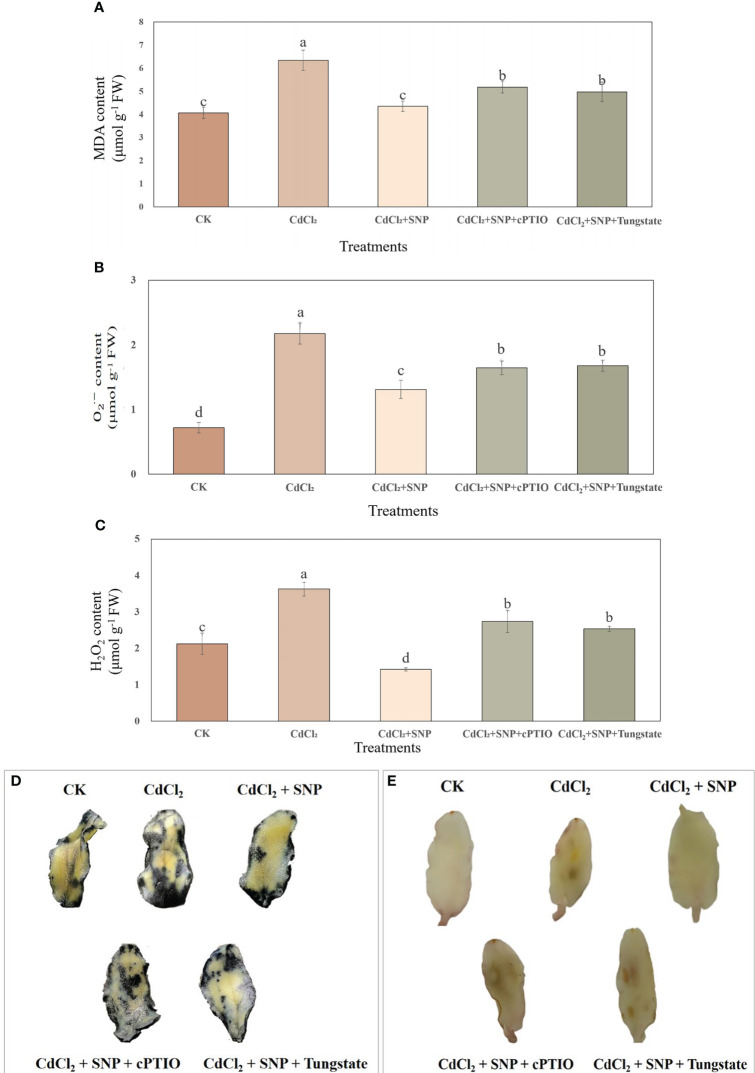
Effect of NO on MDA content (**A**), ${\text{ O}}_{2}^{\;\cdot -}$ content **(B)** and H_2_O_2_ content **(C)** during adventitious root development in cucumber under Cd stress. Explants were incubated for 48 h with distilled water (CK) or 1 μM CdCl_2_, 1 μM CdCl_2_ +10 μM SNP, 1 μM CdCl_2_ + 10 μM SNP + 200 μM cPTIO, 1 μM CdCl_2_ + 10 μM SNP + 200 μM tungstate. Photograph showing NBT **(D)** and DAB **(E)** staining after 48 h of treatments. Bars with different letters are significantly different at *P* < 0.05 according to Duncan’s multiple range test.

The levels of MDA, H_2_O_2_ and ${\text{ O}}_{2}^{\;\cdot -}$ in cucumber explants under Cd stress were measured in our experiment (**Figure 4**). As shown in **Figure 4A**, CdCl_2_ treatment significantly increased MDA content in cucumber explants which compared to that of control. However, exogenous NO significantly decreased MDA level under Cd stress. As compared to Cd stress, the MDA content which treated with NO treatment significantly decreased by 31.4% (**Figure 4A**). However, cPTIO or tungstate treatment significantly elevated the content of MDA compared to CdCl_2_ + NO treatment. Also, CdCl_2_ treatment significantly increased the content of ${\text{ O}}_{2}^{\;\cdot -}$ in cucumber explants. Exogenous NO could reverse the increase in ${\text{ O}}_{2}^{\;\cdot -}$ level which caused by Cd stress (**Figures 4B, D**). As shown in **Figure 4B**, application of NO obviously decreased the ${\text{ O}}_{2}^{\;\cdot -}$ level by 39.6% when compared to CdCl_2_ treatment. However, the content of ${\text{ O}}_{2}^{\;\cdot -}$ in cucumber explants which treated with cPTIO or tungstate was significantly higher than that of CdCl_2_ + NO treatment. Furthermore, the effect of NO treatment on H_2_O_2_ level followed the same pattern as the effect on ${\text{ O}}_{2}^{\;\cdot -}$ level (**Figures 4C, E**). These results indicated that NO could obviously alleviate membrane lipid peroxidation and inhibit the accumulation of ROS, thus reducing oxidative damage and promoting the formation of adventitious root in cucumber under Cd stress.

### 3.5 Effect of NO on the expression level of antioxidant enzymes under Cd stress

**Figure 5:**
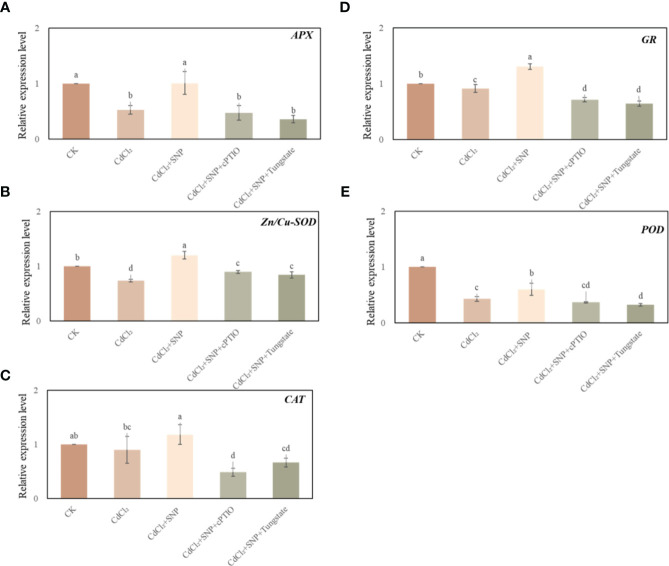
Effect of NO on the expression level of *APX* (**A**), *Zn/Cu-SOD* (**B**), *CAT* (**C**), GR (**D**) and *POD* (**E**) in cucumber explants under Cd stress at 48 (h) Explants were incubated for 2 days with distilled water (CK) or 1 μM CdCl_2_, 1 μM CdCl_2_ +10 μM SNP, 1 μM CdCl_2_ + 10 μM SNP + 200 μM cPTIO, 1 μM CdCl_2_ + 10 μM SNP + 200 μM Tungstate. The values (means ± SE) are the average of three independent experiments. Bars with different letters are significantly different at *P* < 0.05 according to Duncan’s multiple range test.

We further explored the effect of NO on the antioxidant system during adventitious root development under Cd stress. As shown in **Figure 5**, CdCl_2_ treatment has a significant effect on the expression level of antioxidant enzymes during the process of adventitious root formation. Compared to CK treatment, Cd treatment significantly decreased the expression level of ascorbate peroxidase (*APX*), Cu, Zn-superoxide dismutase (*Cu, Zn-SOD*), glutathione reductase (*GR*) and peroxidase (*POD*) (**Figure 5**). However, the expression of these genes in NO treatment was significantly higher than that of Cd stress alone (**Figure 5**). As shown in **Figure 5A**, exogenous NO significantly increased *APX* relative expression by 90.8% compared with Cd treatment alone. Meanwhile, *Zn/Cu-SOD*, *CAT*, *GR* and *POD* relative expression of CdCl_2_ + NO treatment was significantly higher 63.3%, 31.0%, 43.0% and 40.9% than those of Cd treatment, respectively (**Figure 5**). Nevertheless, NO scavengers or inhibitor obviously down-regulated the transcriptional levels of the antioxidant enzymes compared to those of NO treatment under Cd stress (**Figure 5**). Thus, these results might give an exploration of the positive role of NO in hindering ROS production by regulating the transcriptional levels of antioxidant enzymes.

### 3.6 Effect of NO on the expression level of glycolysis-related genes under Cd stress

**Figure 6:**
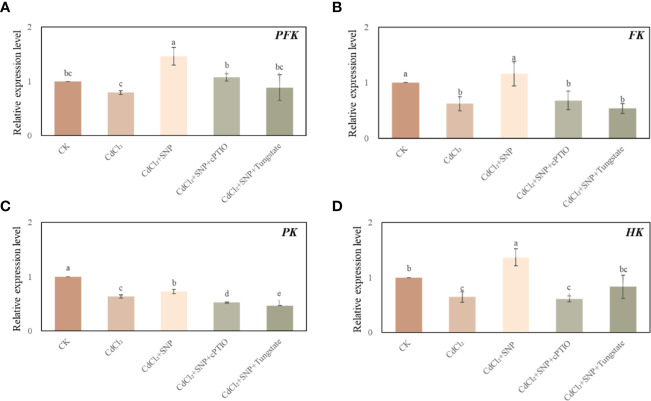
Effect of NO on the expression level of *PFK* (**A**), *FK* (**B**), *PK* (**C**) and *HK* (**D**) in cucumber explants under Cd stress at 48 (h) Explants were incubated for 2 days with distilled water (CK) or 1 μM CdCl_2_, 1 μM CdCl_2_ +10 μM SNP, 1 μM CdCl_2_ + 10 μM SNP + 200 μM cPTIO, 1 μM CdCl_2_ + 10 μM SNP + 200 μM Tungstate. The values (means ± SE) are the average of three independent experiments. Bars with different letters are significantly different at *P* < 0.05 according to Duncan’s multiple range test.

We evaluated the effect of NO on glycolysis pathway during the development of adventitious root in cucumber under Cd stress. As shown in **Figure 6**, compared with CK treatment, CdCl_2_ treatment significantly down-regulated the gene expression levels of *FK, PK* and HK. However, compared to those of Cd treatment, exogenous NO significantly enhanced the expression level of glycolysis-related genes. As shown in **Figures 6A, B**, exogenous NO resulted in a 83.8% increase in *PFK* relative expression and a 87.1% increase in *FK* relative expression compared with Cd treatment alone, respectively. Moreover, Cd treatment decreased *PK* relative expression by 12.4% and caused a 52.7% decrease in *HK* relative expression compared with NO + Cd treatment, respectively (**Figures 6C, D**). On the contrary, NO scavengers or inhibitor obviously reversed the positive effect of NO on regulating the mRNA transcription level of these genes (**Figure 6**). Therefore, NO promoted adventitious rooting under Cd stress through regulating glycolysis-related gene expression.

### 3.7 Effect of NO on the expression level of polyamine enzymes under Cd stress

**Figure 7:**
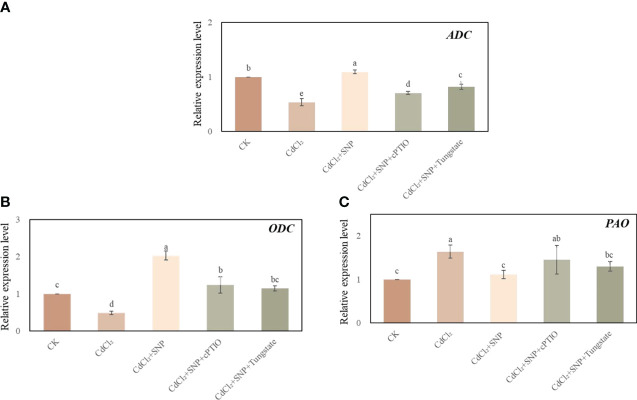
Effect of NO on the expression level of *ADC* (**A**), *ODC* **(B)** and *PAO* (**C**) in cucumber explants under Cd stress at 48 (h) Explants were incubated for 2 days with distilled water (CK) or 1 μM CdCl_2_, 1 μM CdCl_2_ +10 μM SNP, 1 μM CdCl_2_ + 10 μM SNP + 200 μM cPTIO, 1 μM CdCl_2_ + 10 μM SNP + 200 μM Tungstate. The values (means ± SE) are the average of three independent experiments. Bars with different letters are significantly different at *P* < 0.05 according to Duncan’s multiple range test.

Cd stress significantly decreased the expression level of arginine decarboxylase (ADC) and ornithine decarboxylase (ODC) in our experiment (**Figures 7A, B**). As shown in **Figure 7**, compared to CdCl_2_ treatment, the expression level of *ADC* and *ODC* in CdCl_2_ + NO treatment were significantly higher than those of CdCl_2_ treatment alone. Moreover, CdCl_2_ + NO treatment resulted in a 32.2% decrease in *PAO* relative expression compared with Cd treatment. However, NO scavenger or inhibitor treatment could reverse the effect of NO on the expression level of polyamine enzymes (**Figure 7**). These results imply that application of NO could regulate polyamine homeostasis during adventitious root development in response to Cd stress.

## 4 Discussion

Cadmium stress as an environmental factor has a significant impact on the growth and development of plants. It has been confirmed that Cd stress is an important limiting factor for plant growth and development, which inhibits the growth of plants to a certain extent (Azevedo et al., 2012; El Rasafi et al., 2022). As an important signal molecule in plants, NO is involved in a variety of abiotic stress response in plants. In this experiment, we demonstrated that NO could promote the development of adventitious roots of cucumber under Cd stress. In our study, the root number and root length of adventitious roots under Cd stress condition were significantly lower than those of the control (**Figure 1**). These results showed that Cd stress could significantly inhibit the process of adventitious root in cucumber explants. Previous studies found that Cd stress inhibited the adventitious root formation in plants (Li et al., 2019; Gong et al., 2022). However, suitable concentration of NO treatment significantly promoted adventitious root formation under Cd stress (**Figure 1**). Previous studies have shown that NO can resist abiotic stress through protecting cell membrane stability, up-regulating antioxidant enzyme activity and inducing resistance-related gene expression (Fan et al., 2015; Kaya et al., 2015). For example, it has been reported that NO could alleviate Cd toxicity through maintaining the growth regulation and nutritional status in cauliflower (Ma et al., 2022). Also, Zhao et al (2022) found that NO enhanced Cd resistance of *Pleurotus eryngii* through overcoming oxidative damage and regulating short-chain dehydrogenase/reductase famliy. Our results implied that suitable concentration of NO might alleviate the negative effect of Cd stress on the adventitious rooting of cucumber. Moreover, several studies indicated that NO could regulate the growth and development of plant roots, including root elongation, lateral root growth and adventitious root formation (Díaz et al., 2021; Wang et al., 2021; Liu Y.Y. et al., 2022). In our experiment, low concentration of NO significantly alleviated the inhibitory effect of Cd stress on the formation of adventitious root, while high concentration of NO obviously inhibited the occurrence of adventitious root under Cd stress (**Figure 1**). These results showed that NO had a concentration-dependent effect on adventitious root formation under Cd stress. In addition, NO scavengers or inhibitor dramatically reduced the root number and root length of cucumber explants (**Figure 2**), implying that NO plays a vital role in the development of adventitious roots under Cd stress.

Several reports suggested that the endogenous NO accumulation has been implicated as being responsible for the development of adventitious root in plants (Kang et al., 2018; Altamura et al., 2023). Compared to CK treatment, Cd stress significantly decreased endogenous NO levels during adventitious root development (**Figure 3**), implying that Cd stress might lead to a significant reduction of adventitious root formation through inhibiting endogenous NO production. However, exogenous NO significantly increased the endogenous NO production during adventitious root formation under Cd stress (**Figure 3**). Our results are in agreement with previous data on the implication of NO generation during root growth and development under stress conditions. For example, Zhang et al. (2022) suggested that endogenous NO was required for melatonin to stimulate the lateral roots growth of cucumber seedlings under nitrate stress. Moreover, Li et al., (2019) reported that NO significantly elevated endogenous NO level during the adventitious root formation in mung bean hypocotyl under cadmium and osmotic stresses. These observations support the view that NO could promote the formation of adventitious root through enhancing the endogenous NO production under Cd stress.

Excessive ROS results in membrane lipid peroxidation and cell oxidation, causing serious damage to plants (Huang et al., 2019). Meanwhile, MDA level is considered to be an indicator of lipid peroxidation during response to various environmental stresses (Gaweł et al., 2004). Previous study has suggested that Cd treatment caused oxidative stress (He et al., 2014) by increasing the contents of H_2_O_2_ and MDA of rice seedlings. Moreover, under Cd stress, H_2_O_2_ and MDA in wheat plants significantly increased (Kaya et al., 2019). Similarly, in our study, the results showed that CdCl_2_ treatment significantly increased the ROS level and MDA content of cucumber explants during adventitious root formation (**Figure 4**), resulting in oxidative damage (Jaleel et al., 2007), eventually inhibiting adventitious root formation. However, exogenous NO significantly decreased the levels of MDA, H_2_O_2_ and ${\text{ O}}_{2}^{\;\cdot -}$ in cucumber explants to further alleviate the oxidative damage and membrane lipid peroxidation of cucumber explants under Cd stress (Laspina et al., 2005; Jaleel et al., 2007). Previous studies have shown that NO plays a key role in alleviating oxidative stress under Cd stress (Panda et al., 2011; Kaya et al., 2020). For instance, exogenously applied NO significantly reduced oxidative stress and proline content of wheat seedlings under Cd stress (Kaya et al., 2020). Similar to our results, application of NO resulted in an obviously decrease of ${\text{ O}}_{2}^{\;\cdot -}$ , H_2_O_2_ and MDA content to decrease the Cd stress of cauliflower (Ma et al., 2022). These results suggested that NO is involved in ameliorating oxidative impairment under Cd stress. However, the treatment of NO scavengers or inhibitor significantly reversed the positive effect of NO on alleviating the oxidative damage during adventitious root formation under Cd stress (**Figure 4**). These results indicated that NO could significantly reduce the degree of membrane lipid peroxidation and alleviate oxidative stress under Cd stress, thus promoting the occurrence of adventitious roots under Cd stress.

Overproduction of ROS caused oxidative damage, plants need to counteract the toxicity of ROS through a highly efficient antioxidative defense system (Dumanović et al., 2021). At present, plants have effective antioxidant defense mechanisms to alleviate the effects of oxidative stress in plants. Xu et al. (Xu et al., 2008) found that transgenic *Arabidopsis* plants structurally overexpressed peroxisome gene *HvAPX1*, which reduced ROS accumulation and significantly improved the tolerance of *Arabidopsis* plants to Cd stress. In addition, Cd stress mediates the transcriptional expression of *APX*, *GR*, *Cu/ZnSOD* and other related antioxidant enzyme genes in ryegrass, which effectively alleviates the oxidative damage (Luo et al., 2011). Our results showed that Cd stress significantly affected the expression level of antioxidant enzyme genes (**Figure 5**). Moreover, compared to Cd treatment, exogenous NO significantly up-regulated the gene expression levels of these antioxidant enzymes, indicating that NO could alleviate oxidative damage of cucumber explants through enhancing the antioxidant system as well as eliminating excess ROS and MDA (Mostofa et al., 2019; Terrón-Camero et al., 2019). It has been investigated that NO significantly increased the activities of antioxidant enzymes of wheat under Cd stress (Kaya et al., 2019). Moreover, NO have the ability to enhance the antioxidant activities in bamboo plants under Cd stress (Emamverdian et al., 2021). Our present investigation suggested that NO could obviously enhance the plant defense system during adventitious rooting in the response to Cd stress.

The glycolysis process is the basis for controlling carbohydrate metabolism, which also considered to be one of the key pathways for plant respiration (Plaxton, 1996; Sun et al., 2021). In addition, glycolysis has been demonstrated to be involved in the plant response to abiotic stress (Zhang et al., 2011a; Dong et al., 2020; Sun et al., 2021). For example, it has been reported that the increase of PFK and PK activity could enhance the tolerance to salt stress(Zhong et al., 2016). Moreover, the enhancement of the expression level of *PFK*, *PK* and *PEPC* of cucumber leaves allows to convert more carbohydrates and maintain the normal physiological metabolism of cucumber (Zhong et al., 2016). Previous study found that Cd stress caused changes in carbohydrate metabolism, glycolysis and pentose phosphate pathway-related enzymes in pea (Devi et al., 2007). Moreover, Shahid et al (2019) found that Cd treatment significantly inhibited the activities of FK, HK, PFK and PK in potato plants. In our study, we found that Cd stress significantly down-regulated the gene expression levels of key glycolysis enzymes during adventitious root formation (**Figure 6**), indicating that Cd stress may lead to the inhibition of glycolysis pathway and further affect respiratory pathway during the adventitious rooting. However, under Cd stress, NO treatment could significantly up-regulate the gene expression levels of *PFK*, *PK*, *FK* and *HK* (**Figure 6**). In agreement with the present study, NO could obviously elevate the activities of FK to improve the chilling tolerance of banana fruit (Wang et al., 2015). Similarly, Pandey et al (2019) found that NO treatment may up-regulated the expressional level of *HK1-like*, *phosphofructokinase 6-like* and *PK* which involved in glycolysis pathway during seed germination of chickpea. Furthermore, previous study suggested exogenous nitric oxide improved NaCl tolerance by enhancing glycolysis metabolism in barley seedlings (Ma et al., 2021). These results implied that NO plays an essential role in regulating glycolysis metabolism in plant. In the present study, NO may trigger glycolysis metabolic pathway through increasing the gene expression levels of key glycolysis enzymes to produce more energy and activate intermediate metabolism during the process of adventitious root formation under Cd stress, in order to enhance resistance to Cd stress (Shu et al., 2011).

Previous studies found that polyamines (PAs) play an essential role in plant growth and development, as well as response to biotic and abiotic stress (Wimalasekera et al., 2011; Rakesh et al., 2021). In plants, it has been reported that PAs could be produced by ornithine decarboxylase (ODC) or arginine decarboxylase (ADC) pathway, respectively (Groppa and Benavides, 2008). Meanwhile, polyamine oxidase (PAO) plays a major role in mediating PAs degradation in plant (Goyal and Asthir, 2010). In our study, the gene expression of *ADC* and *ODC* was significantly down-regulated in CdCl_2_ treatment, as compared to CK (**Figures 7A, B**). However, exogenous NO obviously enhanced the gene expression of *ADC* and *ODC* which compared with Cd stress, resulting in the accumulation of endogenous polyamine. In addition, removing endogenous NO further implied that NO is involved in PAs accumulation through increasing the expression of *ADC* and *ODC* (**Figures 7A, B**) under Cd stress. Previous studies indicated that PAs metabolism plays a vital role in abiotic stress responses (Gupta et al., 2013). Also, it has been reported that NO could obviously regulate the transcriptional level of polyamine metabolism genes of *Medicago truncatula* (Filippou et al., 2013). Moreover, exogenous NO resulted in cold tolerance by regulating the expression level of *ADC* and *ODC* of tea root (Wang et al., 2020). Furthermore, application of NO could significantly increase the expression of PA biosynthetic enzyme and lower the activity of PAO activity under salt stress (Tailor et al., 2019). In our study, Cd stress remarkably enhanced the expression of *PAO* while a significant decline in *PAO* expression was observed in NO treatment (**Figure 7C**) which may help maintaining PAs levels. These results suggested that NO might enhance abiotic stress tolerance through regulating PAs metabolim. Thus, during adventitious root development under Cd stress, exogenous NO might positively modulate PAs homeostasis through regulating polyamines - related genes expression for adapting to Cd stress condition.

## Conclusion

Exogenous application of NO alleviated Cd damage and promoted the adventitious rooting in cucumber explants under Cd stress. Through further studies, our results suggested that NO could reduce oxidative damage and depress lipid peroxidation through improving the antioxidant capacity of cucumber during adventitious root formation in response to Cd stress. Additionally, NO alleviated the damage of Cd stress on the process of adventitious rooting through regulating glycolysis processes and polyamine homeostasis. Therefore, our study may provide new insights into the positive role of NO in promoting adventitious root development under Cd stress. Taken together, our study provided evidence that NO promoted the adventitious root development under Cd stress in cucumber explants through enhancing antioxidant capability, promoting glycolysis pathway and maintaining polyamine homeostasis. However, the regulatory mechanism underlying NO-induced adventitious root development under Cd stress is complex. Further research should focus on the molecular mechanism of NO-regulated rooting response under Cd stress.

## Data availability statement

The original contributions presented in the study are included in the article/Supplementary Material. Further inquiries can be directed to the corresponding author.

## Author contributions

JY designed the experiments. LN and JY performed the experiments. LN, DW and QC performed data analysis. LN wrote the manuscript. YT, BZ and ZH edited the manuscript. All authors contributed to the article and approved the submitted version.
